# Effectiveness of a Serious Video Game (MOON) for Attention Deficit Hyperactivity Disorder: Protocol for a Randomized Clinical Trial

**DOI:** 10.2196/53191

**Published:** 2024-02-23

**Authors:** Marina Martin-Moratinos, Marcos Bella-Fernández, Maria Rodrigo-Yanguas, Carlos González-Tardón, Aaron Sújar, Chao Li, Ping Wang, Ana Royuela, Pilar Lopez-Garcia, Hilario Blasco-Fontecilla

**Affiliations:** 1 Department of Psychiatry Puerta de Hierro University Hospital Health Research Institute Puerta de Hierro-Segovia de Arana Majadahonda, Madrid, Spain Spain; 2 Faculty of Medicine Autonoma University of Madrid Madrid Spain; 3 Faculty of Psychology Autonoma University of Madrid Madrid Spain; 4 Department of Psychology Pontifical University of Comillas Madrid Spain; 5 Tecnocampus – Universitat Pompeu Fabra Mataro Spain; 6 Department of Computer Engineering Universidad Rey Juan Carlos Móstoles, Madrid Spain; 7 Clinical Biostatistics Unit Consortium for Biomedical Research Network in Epidemiology and Public Health Madrid Spain; 8 Spain Biomedical Research Networking Center for Mental Health Network Madrid Spain; 9 ITA Center Mental Health Specialists Madrid Spain; 10 Health Sciences School and Medical Center Universidad Internacional de La Rioja Madrid Spain

**Keywords:** attention deficit hyperactivity disorder, ADHD, emotional dysregulation, serious video games, virtual reality, cognitive training, music, chess, attention deficit hyperactivity disorder, video game, video games, children, child, adolescents, adolescent, teen, teens, emotional regulation, neurodevelopmental disorder, multimodal treatment, intervention, motivational tools

## Abstract

**Background:**

Attention deficit hyperactivity disorder (ADHD) is the most common neurodevelopmental disorder in childhood and adolescence, with a prevalence of 5% and associated difficulties and worse prognosis if undetected. Multimodal treatment is the treatment of choice. However, sometimes treatment can be insufficient or have drawbacks.

**Objective:**

This study protocol aims to demonstrate the effectiveness of cognitive training through the serious video game The Secret Trail of Moon (MOON) in improving emotional regulation in people with ADHD.

**Methods:**

This is a prospective, unicenter, randomized, unblinded, pre- and postintervention study. The groups will be randomized (MOON vs control) via an electronic case report form. The MOON intervention will be performed 2 times per week for 10 weeks (30 minutes per session). The first 5 weeks (10 sessions) will be conducted face-to-face at the Puerta de Hierro University Hospital, and the remaining weeks will be conducted via the internet at the participants’ homes. The total sample consists of 152 patients aged between 7 and 18 years. All participants have a clinical diagnosis of ADHD under pharmacological treatment. Data collection will be used to obtain demographic and clinical data. The data will be recorded using REDCap. Measures will be made through clinical scales for parents and objective tests of cognitive functioning in patients. Additional information on academic performance will be collected. The study has a power greater than 80% to detect differences. Student *t* test, 2-factor analysis of variance (ANOVA), and Mann-Whitney analyses will be performed according to each variable’s characteristics.

**Results:**

The study was approved by the Research Ethics Committee of the Puerta de Hierro University Hospital on December 14, 2022. As of September 26, 2023, we have enrolled 62 participants, and 31 participants have completed the study. This clinical trial was funded by the Comunidad de Madrid (IND2020/BMD-17544). The approximate completion date is March 2024.

**Conclusions:**

Serious video games such as MOON can be motivational tools that complement multimodal treatment for ADHD.

**Trial Registration:**

ClinicalTrials.gov; NCT06006871; https://clinicaltrials.gov/study/NCT06006871

**International Registered Report Identifier (IRRID):**

DERR1-10.2196/53191

## Introduction

Attention deficit hyperactivity disorder (ADHD) is the most common neurodevelopmental disorder diagnosed in childhood and adolescence, affecting about 5% of people worldwide [1]. In addition to the classical clinical triad of ADHD (inattention, hyperactivity, and impulsivity) [2], the existence of comorbidity, executive dysfunction, or emotional dysregulation can complicate the prognosis [3]. A greater severity of ADHD symptoms is correlated with greater emotional dysregulation in both children [4] and adults [5]. Emotion regulation involves intrinsic and extrinsic processes responsible for managing the appraisal and control of emotional reactions, emotion intensity, temporality, and goal orientation [6]. Emotional dysregulation has a severe impact on social skills, academic performance, and adaptive skills, leading to a higher rate of usage of treatment services [4]. This impact on emotional regulation manifests diversely across broad age ranges in ADHD from children to adults [7-9]. Emotional regulation issues, even in patients aged 5 years and younger, predict inattention later on [10]. Guidelines recommend that emotional dysregulation be treated before the age of 7 years [11]. ADHD is considered a chronic disorder with a high economic cost [12,13], high accident rate, and increased risk of mortality [14].

Multimodal treatment is the most effective treatment for ADHD, and it encompasses pharmacological treatment, psychological treatment, and psychoeducation for parents and teachers. Pharmacological treatment is the treatment of choice in children and adolescents with severe ADHD [15] and the most common treatment in high-income countries [16]. Approximately 70% of patients observe a positive change in symptoms in the short term due to stimulants [17]. However, some drawbacks such as the side effects of pharmacological treatment (insomnia, appetite suppression, and growth retardation) make some parents reluctant [18]. Moreover, medication is more effective for the core symptoms of ADHD than for the bottom-up mechanisms associated with emotional regulation [19]. Cognitive behavioral therapy can help with social skills and problem-solving [20]. However, motivational difficulties in ADHD [3] and low adherence to treatment [18] have led to the development of additional interventions complementing multimodal treatments. Some studies show promising results with cognitive training [21], metacognitive interventions [22], music [23], and video games [24]. However, the overall results show small effect sizes and difficulties in maintaining long-term benefits and transferability to daily life (generalizability and transferability) [25]. More evidence is needed with greater control of risk biases.

Video games provide immersive and engaging entertainment experiences. They have been extended as tools in the field of health (eg, promoting physical exercise through exergames) to others for cognitive training, as discussed in the reviews by Peñuelas-Calvo et al [24] and Rodrigo-Yanguas et al [26]. In particular, a recent systematic review [27] showed the potential use of video games in improving emotional regulation. Using video games for ADHD treatment may be beneficial for several reasons, including their attractiveness, customization to suit the player’s tastes, and immediate reinforcement [28]. Children and adolescents with ADHD have difficulties associated with poor verbal working memory (poor internal language), which can make it difficult for them to perform tasks. In addition, they may be more dependent on external stimuli (ie, they may have low intrinsic motivation), causing them to become bored earlier [3]. Therapeutic interventions based on gamification and video games can be a good strategy to decrease treatment dropout rates by encouraging patients to perform tasks as games.

The Secret Trail of Moon (MOON) is a serious video game based on cognitive training for people with ADHD. It comprises a set of different mini-games focused on enhancing the most affected cognitive abilities in ADHD according to the Executive Functions and Behavioral Inhibition Model [3,29,30]. The games progressively increase in difficulty according to the patient’s needs. MOON was designed by a multidisciplinary team following a user-centered model (usability study) and an iterative redesign process [28,31]. Prior to this clinical trial, we completed another clinical trial. In that prospective, single-center, randomized clinical trial with 3 arms, MOON was tested in clinically stable patients with ADHD (on medication) aged 12 to 22 years (NCT04355065) for 3 months. The 105 patients were randomly assigned to the 3 groups: (1) cognitive training (12 sessions) with face-to-face MOON (n=31; 30); (2) cognitive training with online therapeutic chess (n=24; 23); and (3) control group with telephone monitoring (n=34; 32%). Contrary to our expectations, we did not find any statistically significant improvement in executive functioning, which was the primary outcome. However, we found some improvements in secondary outcomes, such as emotional intelligence, emotional regulation, and performance in the school context in both self-reports and parent reports [32]. Following these encouraging results, we designed the present clinical trial (NCT06006871) with the limitations of the previous clinical trial in mind, namely: (1) increasing sample size; (2) increasing the number of cognitive “doses” (20 sessions) and the access to the platform using virtual reality (VR) and computer interfaces; (3) improving some aspects of the MOON video game, particularly emotional regulation, such as the aesthetics of the virtual environment, the introduction of music [23], rhythm-based gameplay, and motivational elements such as the reward system; and (4) simplifying the design (2 arms, case versus control, instead of 3 arms). The main objective is to test the efficacy of MOON in improving emotional regulation in patients aged 7 to 18 years with a clinical diagnosis of ADHD.

## Methods

### Ethical Considerations

This study was approved by the Research Ethics Committee of the Puerta de Hierro University Hospital on December 14, 2022 (PI 106/22). Authorization from the Spanish Agency of Medicines and Health Products was granted on February 14, 2023 (1061/22/EC-R). Informed consent will be requested from legal guardians and minors protecting their personal data to the provisions of the Organic Law (3/2018) passed on December 5, 2018, regarding personal data protection and guarantee of digital rights.

The Spanish Agency of Medicines and Medical Devices (AEMPS) authorized the clinical research with medical devices lacking Conformité Européenne (CE) marking on February 14, 2023 (1061/22/EC-R). The version approved by both entities was version 4 in the protocol, data collection, and recruitment material. The investigator's brochure was approved in version 3. The ethics committee requested the elimination of the rhythm-based game because it has not been previously tested in patients with ADHD. Monitoring of the clinical trial was deemed necessary.

### Study Design

This is a prospective, unicentric, randomized, unblinded, pre- and postintervention study with a concealed randomization sequence. The groups will be randomized (MOON vs control) via an electronic case report form (CRF). The interventional study model is a parallel assignment. The allocation ratio will be equal in both groups.

### Procedure

**Figure 1:**
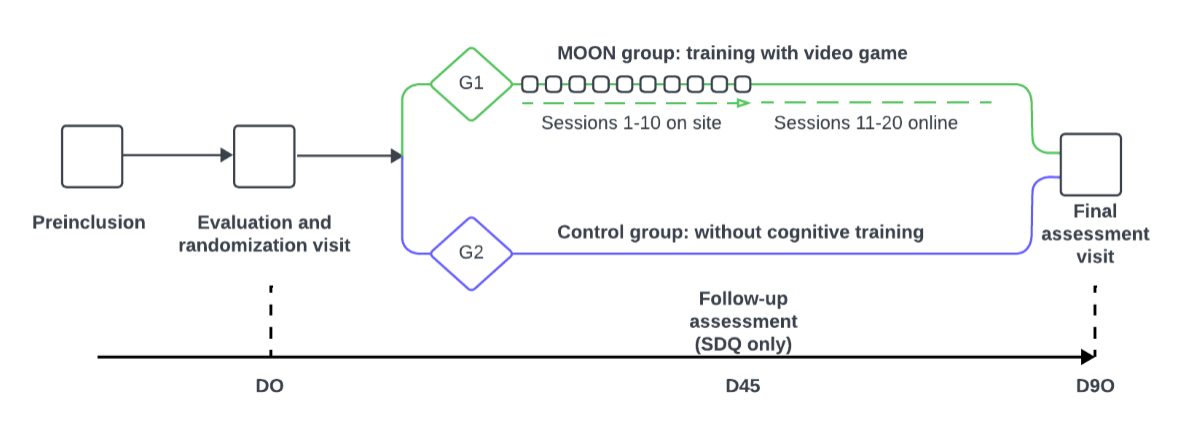
Duration of the research. MOON: The Secret Trail of Moon; SDQ: Strengths and Difficulties Questionnaire.

All participants will be recruited from the child and adolescent psychiatry outpatient clinics of Hospital Universitario Puerta de Hierro Majadahonda. The number of visits varies in each group—the MOON group will have 12 face-to-face visits, while the control group will have only 2 presential visits. The total duration of the research will be 90 days in total (3 months for each participant: 12 weeks including both the pre- and postevaluation periods; Figure 1). Table 1 summarizes all the visits.

**Table 1:** Study flow.

Visit and day	Preinclusion (D1)	Inclusion (D0)	Training (D1-D90)	Final (D90)
Explanation protocol	✓			
CGI^a^ (clinical)	✓			
Randomization		✓		
Informed consent		✓		
Data collection note		✓		
CGI (parents)		✓		✓
SDQ^b^ (parents)		✓	✓	✓
CPRS-HI^c^ (parents)		✓		✓
SNAP-IV^d^ (parents)		✓		✓
SDSC^e^ (parents)		✓		✓
BRIEF-2^f^ (parents)		✓		✓
CPT-3^g^ (patients)		✓		✓
Corsi cubes (patients)		✓		✓
CTMT-2^h^ (patients)		✓		✓
GASA^i^ (patients)		✓		✓
Academic mark		✓		✓
UKU^j^ (patients)			✓	
Satisfaction questionnaire				✓

^a^CGI: Clinical Global Impression Scale.

^b^SDQ: Strengths and Difficulties Questionnaire.

^c^CPRS-HI: Conners Abbreviated Symptom Questionnaire.

^d^SNAP-IV: Swanson, Nolan, and Pelham Rating Scale.

^e^SDSC: Sleep Disturbance Scale for Children for Parents

^f^BRIEF-2: Behavior Rating Inventory Executive Function, Version 2.

^g^CPT-3: Conners Continuous Performance Test, Third Edition.

^h^CTMT-2: Comprehensive Trail-Making Test, Second Edition.

^i^GASA: Game Addiction Scale for Adolescents.

^j^UKU: Udvalg für Kliniske Undersolgester.

**Figure 2:**
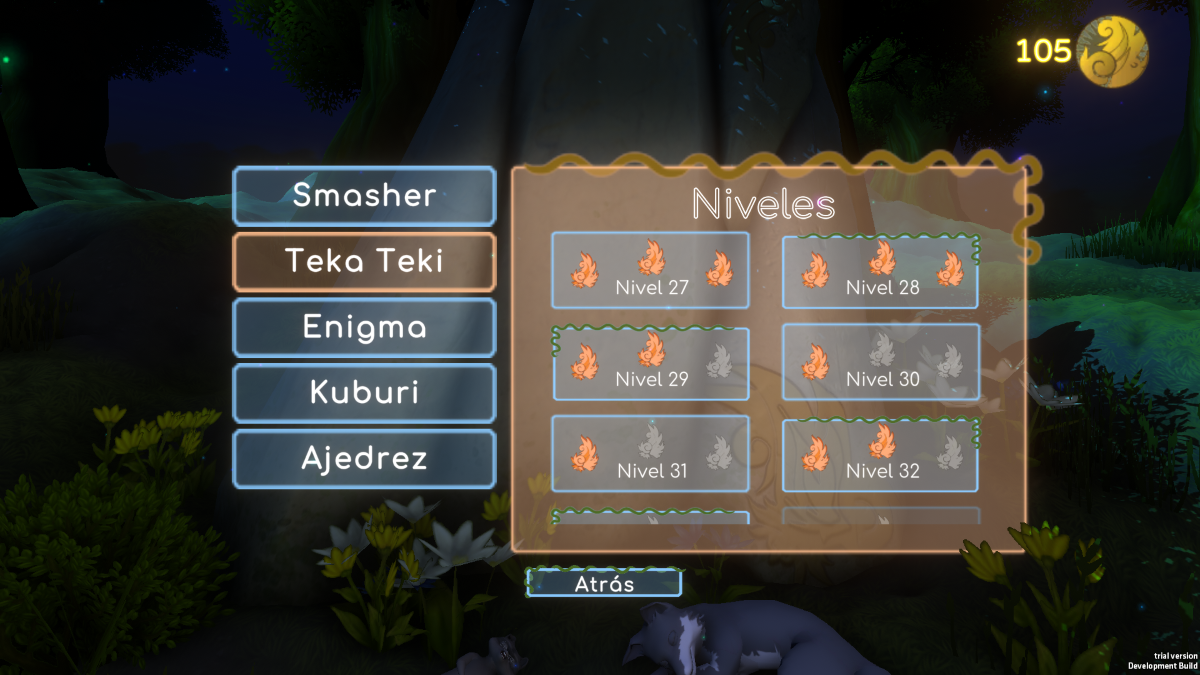
The Secret Trail of Moon (MOON) progress.

**Figure 3:**
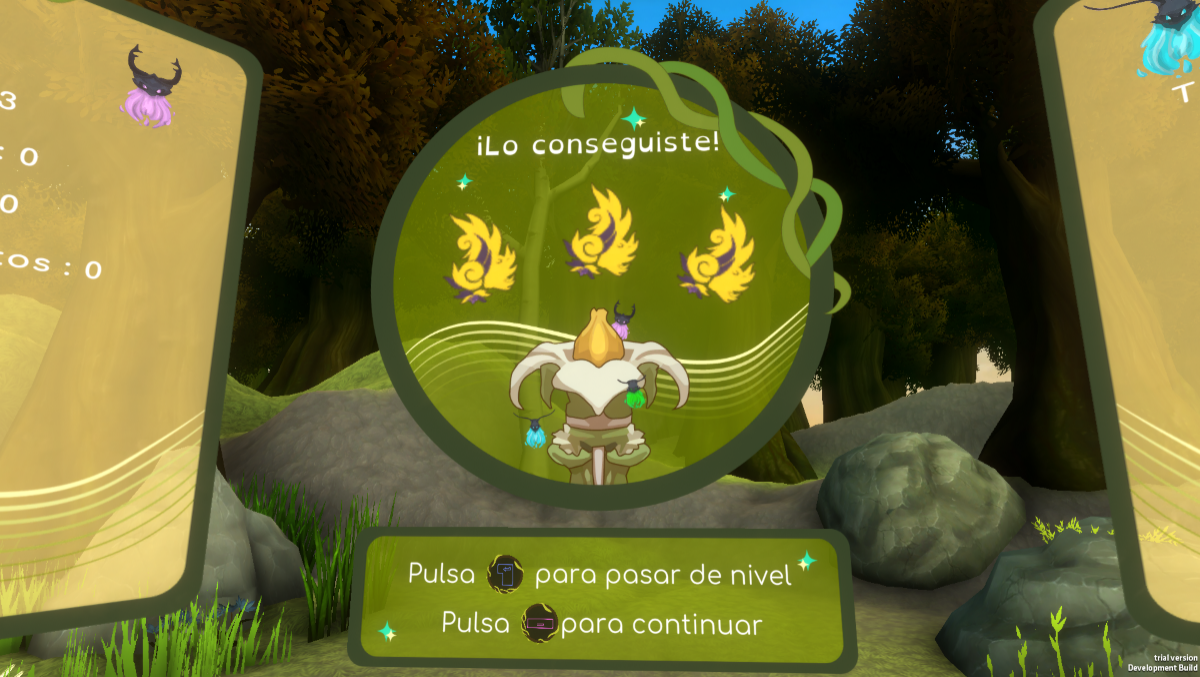
The star system in The Secret Trail of Moon (MOON).

For the MOON group, the total number of sessions with the video game will be 20 (twice a week, adjusted according to the availability of the participants). Participants in the MOON group aged 12 years and over will perform the video game sessions in VR, while those under 12 years will perform the video game sessions on a computer. All participants in the MOON group will perform the cognitive training with MOON following the same order of the games reflected in Table 2. Each session will have a maximum duration of 30 minutes (approximately 20 minutes of MOON gameplay). The first 10 sessions will be held in person at the hospital. For participants over 12 years of age, the researchers will help to manually calibrate the eye distance of the VR headset to adjust the quality of vision. For those under 12 years of age, researchers will perform computer-based support to ensure proper use and understanding of the video game tasks. The researcher will explain the task, adapting the instructions to each participant’s age. Once the researcher is assured of the understanding of the task, the difficulty level will be raised in a personalized way for each participant. Progress is signaled to the player by leveling up, parameters (eg, hits, errors, time taken), and stars earned (0 star=poor performance, must repeat the level; 1 star=acceptable performance; 2 stars=good performance; 3 stars=excellent) (Figures 2 and 3).

**Table 2:** Gameplay order by sessions.

Mode			Onsite at the hospital																			At participants' homes																		
Sessions			1		2		3		4		5		6		7		8		9	10	11			12		13		14		15		16		17		18		19		20
**Games**																																								
	Smasher			1		2								1				2					1						2				1				2			
	Kuburi			2				1				2						1		2							1				1				2					
	Teka-Teki	1						2				1				2					1		2														1		2	
	Enigma	2								1				2						1					2				1		2								1	
	Chess					1				2						1					2				1		2						2		1					

Parents in session 10 will receive a flash drive with the video game and training on how to use it at home. The last 10 sessions will take place at the participants’ homes on their computers and will be monitored via the internet by the researchers. Data collected in the game (eg, completed levels, hits, errors, or reaction times) will be automatically sent to the PlayFab (Microsoft Corp) data server. The parents of MOON group participants will be asked in advance to provide their consent for the collection of these data.

For the control group, participants will be followed up with weekly calls to parents during this period. During this follow-up, psychoeducational support about ADHD will be provided to the parents.

### Hypothesis

Our main hypothesis is that patients with ADHD using MOON will improve their emotional regulation more than the control group. The efficacy will be evaluated by measuring the change produced from the baseline evaluation (day 0) and the final evaluation visit (day 90), aiming for a decrease of 3 to 4 points in the global score of the Strengths and Difficulties Questionnaire (SDQ) filled by the patient’s parents.

As secondary hypotheses, the difference with the control group in measures such as ADHD core symptomatology, cognitive abilities, and academic performance will be assessed. Our secondary hypotheses are listed in Textbox 1.

**Secondary hypotheses of the study.**

- H2: Patients with Attention deficit hyperactivity disorder (ADHD) using The Secret Trail of Moon (MOON) video game will improve in core ADHD symptoms compared with the control group.
- H3: Patients with ADHD using MOON will improve their cognitive functioning compared to the control group.
- H4: Patients with ADHD using MOON will improve in academic performance with respect to the control group.
- H5: A change in platform (face-to-face, internet) will not entail differences in emotional regulation.
- H6: There will be no clinically meaningful side effects associated with the video game.

### Participants

#### Sample

A total of 152 patients (76 cases versus 76 controls) with a clinical diagnosis of ADHD according to the DSM-V (Diagnostic and Statistical Manual of Mental Disorders, Fifth Edition) will be enrolled. All participants will have a clinical diagnosis of ADHD, take ADHD medication, be clinically stable, and have a Clinical Global Impression (CGI) score between 3 and 6 before entering the trial. Medication will not change during the investigation unless changes are required for clinical reasons. A 15% loss rate is expected. The inclusion and exclusion criteria are summarized in Textbox 2.

**Participant inclusion and exclusion criteria.**

Inclusion criteria:

- Age 7 to 17 years (may turn 18 during the study)
- Clinical diagnosis of attention deficit hyperactivity disorder (ADHD) in any presentation
- Taking pharmacological treatment for ADHD
- Ability to follow verbal instructions
- Ability to play a video game (not necessary to play regularly)
- Clinically stable, with ADHD symptomatology severity based on a clinician-assessed Clinical Global Impression (CGI) score between 3 and 6

Exclusion criteria:

- Patients with severe symptoms (> or equal to 5 CGI) or very mild symptoms (CGI < or equal to 1)
- Patient at risk of suicide (according to the clinical judgment of the professional in charge of the patient)
- Motor difficulties that prevent playing the video game
- Participation in other similar studies
- Intention to initiate any psychotherapeutic treatment (including cognitive-behavioral therapy) in the next 3 months of the course of participation in the clinical trial

#### Randomization and Masking

To warrant clinical trial randomization, a random block sequence of 76 “1” (experimental) and 76 “2” (control) in 4 blocks of 38 numbers (19 “1” and 19 “2”) will be generated using the R program (R Foundation for Statistical Computing) by author MBF [33]. The sequence will be unknown to the recruiters [34].

In the preinclusion phase, patients will be informed about the research by the principal investigator (author HBF) and their suitability will be assessed according to the inclusion and exclusion criteria (Textbox 2). Subsequently, another author (MMM) will explain the research procedure in detail and summon the prerecruited participants. Once the consent form is signed, they will be randomly assigned by blocks using the electronic CRF with a ratio of 1:1. Randomization of the groups (MOON vs control) will be performed by the electronic CRF REDCap.

### Materials

#### Hardware

**Figure 4:**
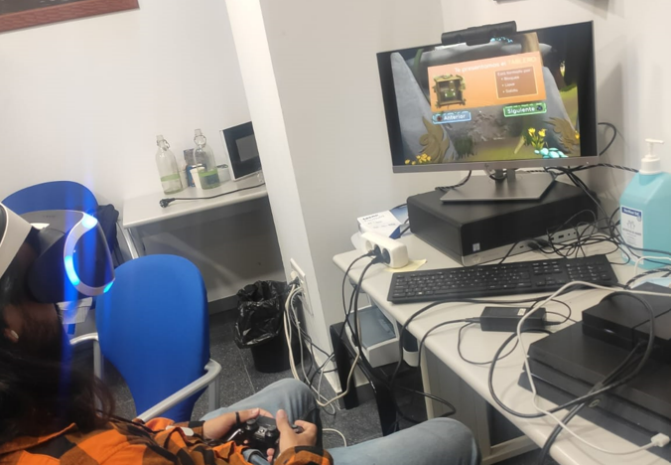
User playing The Secret Trail of Moon (MOON).

Materials to be used for this study include the video game (MOON) itself, VR goggles, PlayStation 4 (Sony Group Corp) controllers, test consoles, monitor screens, and headsets. The VR software runs on a PlayStation 4 test device (Figure 4). A computer with an internet connection will also be used in this study to ensure the proper transmission of game data to the PlayFab server.

##### Software: Video Game

MOON has been designed to be a therapeutic cognitive training video game for patients with ADHD. This serious video game can be used both on a computer and s PlayStation 4 VR console. The use of VR allows a more focused approach to the task and a greater impact on the participants’ motivation. The use of a computer allows more accessibility. The game is set in a natural environment, potentially beneficial for people with ADHD with animals that accompany the player throughout the training session [25]. Various games or tasks are incorporated into the game design with the purpose of training the most affected cognitive functions in ADHD. MOON has 5 game mechanics. These are as follows: (1) SMASHER (sustained attention and inhibitory control) is a task based on the Continuous Performance Test, Third Version (CPT-3) [35], with a 2-item sequence similar to Sustained Attention Task in Childhood Test (CSAT) [36]; (2) in TEKATEKI (planning), the participant is asked to perform a minimum number of possible movements (based on the *Tower of Hanoi*); (3) in ENIGMA (working memory), the *span* of items to remember increases according to the difficulty curve [37]; (4) KUBURI (visuospatial ability) is a 3D cube rotation task; and (5) CHESS (reasoning) consists of tasks related to the rules of chess.

### Psychometric Assessments

#### Preinclusion

##### CGI Scale for Clinicians

Symptom severity will be measured with the CGI scale [38] (approximately 1-minute duration). ADHD symptomatology severity based on clinician-assessed CGI score (between 3 and 6) for the inclusion criteria.

#### Clinical Anamnesis

The clinical anamnesis includes demographic, clinical, school, and medical history data.

#### SDQ For Parents

The SDQ has 25 items (approximately a 5-minute duration) [39]. It measures (1) emotional symptoms, (2) behavioral challenges, (3) hyperactivity, (4) conflicts/issues with peers, and (5) prosocial behavior. A decrease of 3 to 4 points in the postassessment visit (D70) concerning the preassessment visit (D0) will be considered an improvement in emotional regulation.

#### The Swanson, Nolan, and Pelham Rating Scale for Parents

The main ADHD symptomatology (inattention, hyperactivity, impulsivity) will be measured using subjective scales for parents. The Swanson, Nolan, and Pelham Rating Scale (SNAP-IV) is an 18-item questionnaire for assessing ADHD symptoms [40,41]. It has a Likert scale ranging from 0 to 4 (approximately 5 minutes long), among which 9 items assess attention deficit and the other 9 items assess hyperactive impulsive component. The cut-off point for attention deficit is 1.78 for parents. For hyperactivity-impulsivity, the cut-off point is 1.44 for parents.

#### The Conners Abbreviated Symptom Questionnaire for Parents

The Conners Abbreviated Symptom Questionnaire (CPRS-HI) helps assess patients with ADHD [42,43]. It is a 10-item questionnaire with a Likert scale of 0 to 3 (approximately 2 minutes long). This revised and abbreviated version of the Conners scale is designed to be answered by parents of children aged 6 to 18 years. It contains Likert-type responses (0= not true at all/never; 1= just a little true/occasionally; 2= Pretty much true/often; 3=very much true/very often). The cut-off points are divided by sex. For boys, a score above 16 is suspected ADHD, while for girls, a suspected diagnosis of ADHD is above 12 points.

#### CGI for Parents

Symptom severity will be measured with the CGI adapted for parents (approximately 1-minute duration) consisting of a “thermometer” with a Likert scale ranging between 1 and 10 [44].

#### Behavior Rating Inventory Executive Function 2 Questionnaire for Parents

The Behavior Rating Inventory Executive Function, Version 2 (BRIEF-2) is a questionnaire designed to evaluate executive functions in children and adolescents [45]. It consists of 63 items with 3 answer options (never, sometimes, and frequently). Its correction provides 4 general indices: emotional regulation, cognitive regulation, behavioral regulation, and global index of executive function. It also provides 2 second-order factors and a general index.

#### CPT-3 for Patients with ADHD

The CPT-3 is a screening task for ADHD in addition to measuring sustained attention, impulse control, and processing speed [35]. It is a computerized, standardized, and validated application test for different age and gender groups. The task consists of pressing a button each time a letter (target) appears on the screen, except for the letter X (nontarget), which must not be pressed. The duration is 14 minutes, and the presentation interval between letters varies (1, 2, and 4 seconds). The test provides results on hits, errors of omission (undetected target) or commission (reacted nontarget), which are considered a measure of impulsivity. In addition, CPT3-3 provides information on hit mean reaction time and variability.

#### Sleep Disturbance Scale for Children for Parents

The Sleep Disturbance Scale for Children (SDSC) is a 26-item questionnaire with a Likert scale ranging between 1 and 5 (1: never; 5: always). It has an approximate duration of 5 minutes [46].

#### Corsi Cubes for Patients With ADHD

Corsi cubes are used to measure visuospatial working memory [37,47].

#### Comprehensive Trail-Making Test, Second Edition for Patients With ADHD

**Figure 5:**
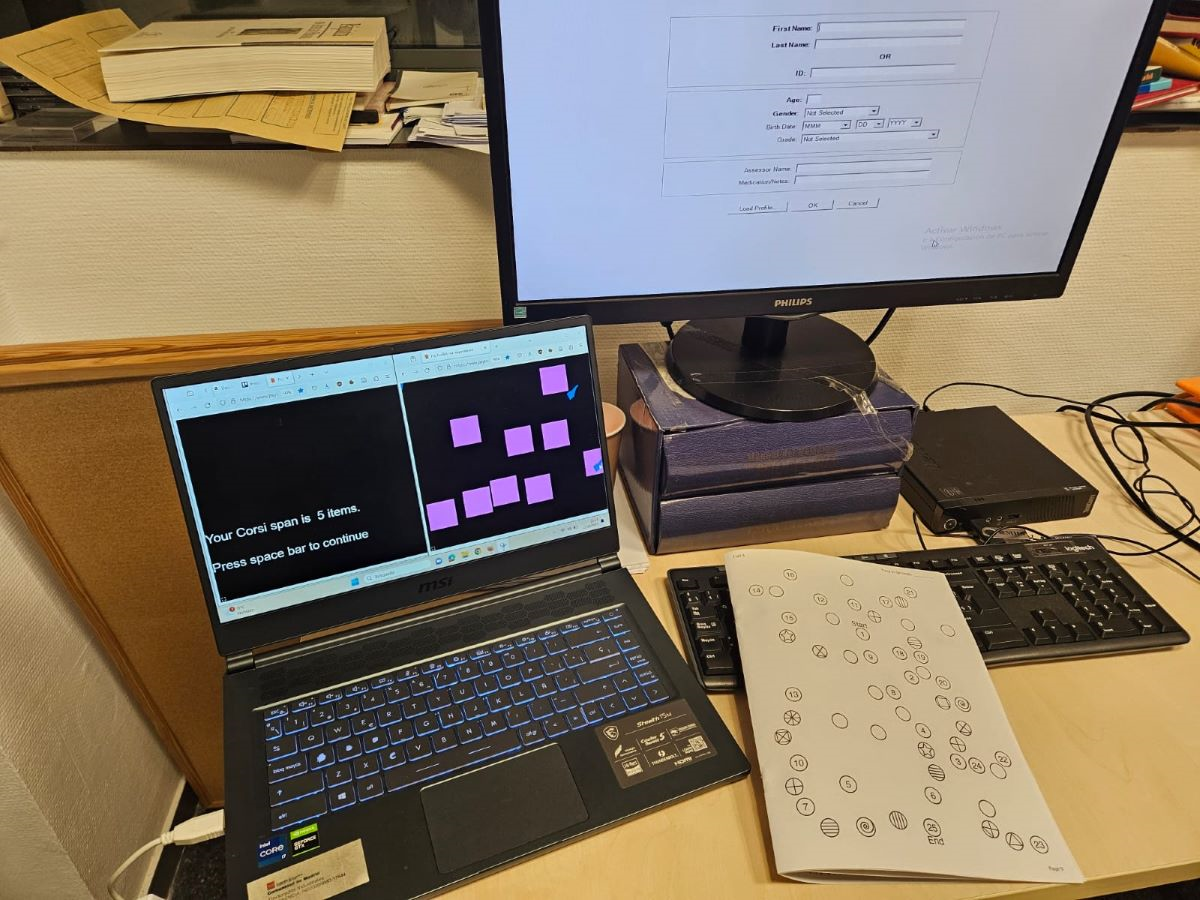
Psychometric assessments: Corsi Cubes, Continuous Performance Test, Third Version (CPT-3), and Comprehensive Trail-Making Test, Second Edition (CTMT-2).

The Comprehensive Trail-Making Test, Second Edition (CTMT-2) is used to measure cognitive flexibility, with 3 indexes: inhibitory control, task switching, and total index (Figure 5) [48].

#### Udvalg für Kliniske Undersogelser for Patients With ADHD

Udvalg für Kliniske Undersogelser (UKU) is a tool designed to evaluate possible secondary symptoms that happen during the week [49]. This scale is not included in the assessment phases. Only the participants playing VR will have to complete the scale after each cognitive training session.

#### Game Addiction Scale for Adolescents

The Game Addiction Scale for Adolescents (GASA) is a 7-item questionnaire to assess video game addiction [50,51].

#### Academic Performance

Information about academic performance will be obtained via the patients’ grades.

### Interventions

After the initial evaluation, 2 groups randomized by blocks will be formed. Group 1 (MOON) will receive personalized cognitive training with a video game in person and via the internet. Psychoeducational support on ADHD to parents will be provided during the research (n=76; 50%).

Group 2 (control) will receive the usual pharmacological treatment (without cognitive training intervention). Psychoeducational support on ADHD will be provided to the parents during the research (n=76; 50%).

### Analyses

#### Sample Size Calculation

Based on similar investigations with hypotheses that are centered around the administration of the SDQ [52], our sample size calculation shows the need to perform the study on approximately 152 participants with a diagnosis of ADHD to cover most of the possibilities with a Cronbach α of 0.05 and a statistical power of 80%, taking into account a 15% dropout rate. We performed the sample size calculation to contrast a mean in the control group of 11 points on the SDQ global score versus a 2-point mean drop (symptomatologic improvement) in the experimental group with a pooled SD of 4. This estimate covers other less conservative situations, such as a difference of up to 4 points.

#### Study Population

The intention-to-treat (ITT) analysis population is defined as participants who are randomized and receive at least 10 sessions, and the per-protocol population is defined as participants who will complete 100% of the training. All analyses will be performed on the premise of the ITT analysis.

A 2-way analysis of variance (ANOVA) will be performed, with time (pre and post) and group (experimental vs control) as factors and each measure as dependent variable. The difference between the study groups will be considered significant when P<.05.

#### Demographic and Baseline Characteristics

A data collection notebook will be used to obtain demographic and clinical data. The data will be recorded with electronic CRF (REDCap). Measures to answer the hypotheses will be made through clinical scales for parents and objective tests of cognitive abilities in patients. Additional information on academic performance will be collected.

#### Primary End Point Analysis

The main objective is to test the efficacy of the cognitive training treatment (MOON) in improving emotional regulation in patients aged 7 to 18 years who have a clinical diagnosis of ADHD. The effectiveness is evaluated by measuring the change produced between the baseline evaluation (day 0) and the final evaluation visit (day 90) in the decrease of 3 to 4 points in the global SDQ score evaluated by the parents. A decrease of 3 to 4 points on the postassessment visit (D70) concerning the preassessment visit (D0) will be considered an improvement in emotional regulation based on other similar studies [52].

Subsequently, 3 parent SDQ measures will be assessed: initial assessment (D0), midterm assessment (D45), and final assessment (D90) to evaluate hypothesis 5 (switching from face-to-face to web-based methods does not lead to differences in emotional regulation).

#### Secondary End Point Analysis

Two subjective scales will be used to assess hypothesis 2 (patients with ADHD using MOON will improve in ADHD symptomatology relative to the control group). The main ADHD symptomatology (inattention, hyperactivity, and impulsivity) will be measured with (1) SNAP, an 18-item questionnaire with a Likert scale ranging between 0 and 4 (approximately 5 minutes long) and (2) CPRS-HI, a 10-item questionnaire with a Likert scale ranging between 0 and 3 (approximately 2 minutes long). Symptom severity will be measured with the CGI adapted for parents (approximately 1-minute duration) consisting of a “thermometer” with a Likert scale ranging between 1 and 10.

Relative to hypothesis 3 (Patients with ADHD using MOON will improve their cognitive abilities more than the control group), executive dysfunction will be evaluated through subjective scales for parents and objective tests for patients. The subjective test for parents will be performed with the BRIEF-2 questionnaire. There will be three objective tests for the patients: (1) CPT-3, which is usually used to screen for ADHD in addition to measuring sustained attention, impulse control, and processing speed; (2) Corsi cubes, which are used to measure visuospatial working memory; and (3) CTMT-2, which is used to measure cognitive flexibility using 3 indexes, namely, inhibitory control, task switching, and total index.

Regarding hypothesis 4 (patients with ADHD using MOON will improve in academic performance relative to the control group), grades will be collected for each subject on the quarterly school report card immediately preceding (pre) and following (post) treatment.

#### Safety and Adherence Analysis

Additional measures have been taken concerning the previous clinical trial [32] to assess the occurrence of side effects. Hypothesis 6 (there will be no side effects associated with the video game) is related to this aspect. To mitigate the possibility of motion sickness due to VR use, the UKU test will be used. In the previous clinical trial, nonsignificant effects related to sleep were observed; therefore, in this clinical trial, the SDSC scale will be used to assess this symptomatology in depth. Since people with ADHD are more prone to addictive behaviors related to video games [53], two main precautions will be taken: (1) adapting the game design so as not to encourage addictive behaviors [28] and (2) collecting additional information with the GASA test.

## Results

The clinical trial is funded by the Community of Madrid (2020 Industrial Doctorates IND2020/BMD-17544). The expected date of data collection was between May 2023 and January 2024. The approximate completion date is March 2024. As of September 26, 2023, we have enrolled 62 participants. A total of 31 (20%) participants completed the study. The analysis of the total results is expected to be published in May 2024.

## Discussion

ADHD is a neurodevelopmental disorder with great variability. Some authors associate the symptoms of inattention with a disorder of “cool” executive function (dorsolateral prefrontal cortex pathway), while the symptoms of hyperactivity and impulsivity are associated with deficits of “hot” executive function (orbital and medial prefrontal cortex pathway) [54]. According to Gross [55,56], emotional regulation is a process whereby people modulate their emotions using strategies such as suppression, strategy modulation, or reappraisal. This process requires an interaction of executive functions with emotional regulation. In relation to these intrinsic-extrinsic processes, children with ADHD compared with typically developing children tend to overestimate their performance in different domains (social, school, and behavioral). Some authors call this term “positive illusory bias” (PIB), with consequent disparity between children’s self-reports and parents’ assessments [22,57].

The MOON video game was developed as a form of cognitive training to improve cognitive abilities, such as sustained attention, inhibitory control, working memory, visuospatial ability, reasoning, and planning [30,31]. However, after conducting the first clinical trial, our main hypothesis of improving executive functions measured with the BRIEF-2 test was not fulfilled [32]. Nevertheless, we did find improvements in emotional domains compared to the control group both in self-reports and parent reports (the most relevant being emotional intelligence, emotional regulation, and performance in the school context). Accordingly, in this second clinical trial, we wanted to replicate and extend our previous positive findings on different measures of emotion regulation. The global SDQ scale we used includes measurements of emotional symptoms, behavioral problems, hyperactivity, problems with peers, and prosocial behavior [39,52].

We believe that any improvement found in measures should be transferred or generalized to daily life. Thus, in this study, we will measure academic performance to assess whether the improvement in cognitive domains includes a transfer to other domains such as arithmetic or reading.

Additional measures have been adopted. Improvements have been added to make the video game more attractive (music, aesthetics, and rewards). To avoid the presence of PIB causing questionnaires to be completed by ”bad informants,” objective tasks, including CPT-3 [35], Corsi cubes [37], and CTMT-2 [48], were proposed for participants in this second clinical trial. To mitigate THE possible excessive use of video games or addiction, the video game design was controlled [28], and the GASA test will be incorporated [50,51].

However, this clinical trial has important limitations. First, a blinded study is not possible. Second, cognitive domains are difficult to measure in the absence of unified questionnaires; in fact, some laboratory measures related to executive function are moderately related to the main symptoms of ADHD and their impact on daily life. As for the video game, it was not possible to incorporate all the improvements designed. Despite the incorporation of children under 12 years of age into this clinical trial, it is possible that some difficulty levels are not well adjusted due to not having been able to perform a usability study prior to this new clinical trial. Moreover, typical of ADHD symptomatology, the decay of the video game sessions—especially those sessions conducted at home without researcher supervision—may occur. Participants could lose motivation for the treatment as the same game is played for a long time [58].

Video games can be a potential tool to improve different skills, such as emotional regulation [27]. In people with ADHD, it is especially important to incorporate motivational tools complementary to multimodal treatment that can facilitate treatment adherence.

## Abbreviations

ADHD: attention deficit hyperactivity disorder
BRIEF-2: Behavior Rating Inventory Executive Function, Version 2
CGI: Clinical Global Impression
CPRS-HI: Conners Abbreviated Symptom Questionnaire
CPT-3: Continuous Performance Test, Third Edition
CRF: case report form
CSAT: Sustained Attention Task in Childhood Test
CTMT-2: Comprehensive Trail-Making Test, Second Edition
DSM-V: Diagnostic and Statistical Manual of Mental Disorders, Fifth Edition
ITT: intention to treat
MOON: The Secret Trail of Moon
PIB: positive illusory bias
SDQ: Strengths and Difficulties Questionnaire
SNAP-IV: Swanson, Nolan, and Pelham Rating Scale
VR: virtual reality

## References

[1] Polanczyk GV, Salum GA, Sugaya LS, Caye A, Rohde LA (2015). Annual research review: A meta-analysis of the worldwide prevalence of mental disorders in children and adolescents. J Child Psychol Psychiatry.

[2] American Psychiatric Association (2013). Diagnostic and Statistical Manual of Mental Health Disorders, 5th Edition.

[3] Barkley R (2015). Attention-Deficit Hyperactivity Disorder: A Handbook for Diagnosis and Treatment, 4th Edition.

[4] Baykal S, Nalbantoglu A (2019). An examination of emotion regulation and associated factors in attention deficit-hyperactivity disorder. Konuralp Medical Journal.

[5] Rüfenacht Eva, Euler S, Prada P, Nicastro R, Dieben K, Hasler R, Pham E, Perroud N, Weibel S (2019). Emotion dysregulation in adults suffering from attention deficit hyperactivity disorder (ADHD), a comparison with borderline personality disorder (BPD). Borderline Personal Disord Emot Dysregul.

[6] Thompson RA (1994). Emotion regulation: a theme in search of definition. Monogr Soc Res Child Dev.

[7] Graziano PA, Garcia A (2016). Attention-deficit hyperactivity disorder and children's emotion dysregulation: A meta-analysis. Clin Psychol Rev.

[8] Groves NB, Wells EL, Soto EF, Marsh CL, Jaisle EM, Harvey TK, Kofler MJ (2022). Executive functioning and emotion regulation in children with and without ADHD. Res Child Adolesc Psychopathol.

[9] Christiansen H, Hirsch O, Albrecht B, Chavanon M (2019). attention-deficit/hyperactivity disorder (ADHD) and emotion regulation over the life span. Curr Psychiatry Rep.

[10] Brocki KC, Forslund T, Frick M, Bohlin G (2019). Do individual differences in early affective and cognitive self-regulation predict developmental change in ADHD symptoms from preschool to adolescence?. J Atten Disord.

[11] Overgaard KR, Aase H, Torgersen S, Reichborn-Kjennerud T, Oerbeck B, Myhre A, Zeiner P (2014). Continuity in features of anxiety and attention deficit/hyperactivity disorder in young preschool children. Eur Child Adolesc Psychiatry.

[12] Doshi JA, Hodgkins P, Kahle J, Sikirica V, Cangelosi MJ, Setyawan J, Erder MH, Neumann PJ (2012). Economic impact of childhood and adult attention-deficit/hyperactivity disorder in the United States. J Am Acad Child Adolesc Psychiatry.

[13] Holden SE, Jenkins-Jones S, Poole CD, Morgan CL, Coghill D, Currie CJ (2013). The prevalence and incidence, resource use and financial costs of treating people with attention deficit/hyperactivity disorder (ADHD) in the United Kingdom (1998 to 2010). Child Adolesc Psychiatry Ment Health.

[14] Dalsgaard S, Østergaard SD, Leckman JF, Mortensen PB, Pedersen MG (2015). Mortality in children, adolescents, and adults with attention deficit hyperactivity disorder: a nationwide cohort study. Lancet.

[15] Faltinsen E, Zwi M, Castells X, Gluud C, Simonsen E, Storebø OJ (2019). Updated 2018 NICE guideline on pharmacological treatments for people with ADHD: a critical look. BMJ Evid Based Med.

[16] Banaschewski T, Coghill D, Santosh P, Zuddas A, Asherson P, Buitelaar J, Danckaerts M, Döpfner M, Faraone SV, Rothenberger A, Sergeant J, Steinhausen H, Sonuga-Barke EJ, Taylor E (2008). [Long-acting medications for the treatment of hyperkinetic disorders - a systematic review and European treatment guidelines. Part 2: a quantitative evaluation of long-acting medications]. Z Kinder Jugendpsychiatr Psychother.

[17] Sandler AD (2002). Practice parameter for the use of stimulant medications in the treatment of children, adolescents and adults. J Dev Behav Pediatr.

[18] Toomey SL, Sox CM, Rusinak D, Finkelstein JA (2012). Why do children with ADHD discontinue their medication?. Clin Pediatr (Phila).

[19] Lenzi F, Cortese S, Harris J, Masi G (2018). Pharmacotherapy of emotional dysregulation in adults with ADHD: A systematic review and meta-analysis. Neurosci Biobehav Rev.

[20] Dalrymple RA, McKenna Maxwell L, Russell S, Duthie J (2020). NICE guideline review: Attention deficit hyperactivity disorder: diagnosis and management (NG87). Arch Dis Child Educ Pract Ed.

[21] Robledo-Castro C, Lerma-Castaño PR, Bonilla-Santos G (2023). Effect of cognitive training programs based on computer systems on executive functions in children with ADHD: a systematic review. J Atten Disord.

[22] Basile A, Toplak ME, Andrade BF (2021). Using metacognitive methods to examine emotion recognition in children with ADHD. J Atten Disord.

[23] Martin-Moratinos M, Bella-Fernández M, Blasco-Fontecilla H (2023). Effects of music on attention-deficit/hyperactivity disorder (ADHD) and potential application in serious video games: systematic review. J Med Internet Res.

[24] Peñuelas-Calvo I, Jiang-Lin LK, Girela-Serrano B, Delgado-Gomez D, Navarro-Jimenez R, Baca-Garcia E, Porras-Segovia A (2022). Video games for the assessment and treatment of attention-deficit/hyperactivity disorder: a systematic review. Eur Child Adolesc Psychiatry.

[25] Rabipour S, Raz A (2012). Training the brain: fact and fad in cognitive and behavioral remediation. Brain Cogn.

[26] Rodrigo-Yanguas M, González-Tardón C, Bella-Fernández M, Blasco-Fontecilla H (2022). Serious Video Games: Angels or Demons in Patients With Attention-Deficit Hyperactivity Disorder? A Quasi-Systematic Review. Front Psychiatry.

[27] Villani D, Carissoli C, Triberti S, Marchetti A, Gilli G, Riva G (2018). Videogames for emotion regulation: a systematic review. Games Health J.

[28] Sújar Aarón, Martín-Moratinos Marina, Rodrigo-Yanguas M, Bella-Fernández Marcos, González-Tardón Carlos, Delgado-Gómez David, Blasco-Fontecilla H (2022). Developing serious video games to treat attention deficit hyperactivity disorder: tutorial guide. JMIR Serious Games.

[29] Brown TE (2008). ADD/ADHD and impaired executive function in clinical practice. Curr Psychiatry Rep.

[30] Rodrigo-Yanguas M, Martín-Moratinos M, Tardón C, Blasco-Fontecilla H (2020). Virtual reality and chess. A video game for cognitive training in patients with ADHD. Proceedings of the VI Congreso de la Sociedad Española para las Ciencias del Videojuego.

[31] Rodrigo-Yanguas M, Martin-Moratinos M, Menendez-Garcia A, Gonzalez-Tardon C, Royuela A, Blasco-Fontecilla H (2021). A virtual reality game (The Secret Trail of Moon) for treating attention-deficit/hyperactivity disorder: development and usability study. JMIR Serious Games.

[32] Rodrigo-Yanguas M, Martín-Moratinos M, González-Tardón C, Sanchez-Sanchez F, Royuela A, Bella-Fernández M, Blasco-Fontecilla H (2023). Effectiveness of a personalized, chess-based training serious video game in the treatment of adolescents and young adults with attention-deficit/hyperactivity disorder: randomized controlled trial. JMIR Serious Games.

[33] (2021). R: A language and environment for statistical computing. The R Project for Statistical Computing.

[34] Schulz KF, Grimes DA (2002). Allocation concealment in randomised trials: defending against deciphering. Lancet.

[35] Conners K (2014). Conners Continuous Performance Test 3rd Edition (CPT 3).

[36] Servera M, Llabrés J (2004). CSAT Tarea de atención sostenida para niños. Hogrefe.

[37] Corsi PM (1972). Human Memory and the Medial Temporal Region of the Brain. Thesis.

[38] Busner J, Targum SD (2007). The clinical global impressions scale: applying a research tool in clinical practice. Psychiatry (Edgmont).

[39] Goodman R (1997). The Strengths and Difficulties Questionnaire: a research note. J Child Psychol Psychiatry.

[40] Swanson JM, Schuck S, Porter MM, Carlson C, Hartman CA, Sergeant JA, Clevenger W, Wasdell M, McCleary R, Lakes K, Wigal T (2012). Categorical and dimensional definitions and evaluations of symptoms of ADHD: history of the SNAP and the SWAN rating scales. Int J Educ Psychol Assess.

[41] Grañana Nora, Richaudeau A, Gorriti CR, O'Flaherty M, Scotti ME, Sixto L, Allegri R, Fejerman N (2011). Assessment of attention deficit hyperactivity: SNAP-IV scale adapted to Argentina. Rev Panam Salud Publica.

[42] Parker J, Sitarenios G, Conners C (2016). Abbreviated Conners' Rating Scales revisited: A confirmatory factor analytic study. J Atten Disord.

[43] Conners C, Sitarenios G, Parker J, Epstein J (1998). The revised Conners' Parent Rating Scale (CPRS-R): factor structure, reliability, and criterion validity. J Abnorm Child Psychol.

[44] Busner J, Targum S (2007). The clinical global impressions scale: applying a research tool in clinical practice. Psychiatry (Edgmont).

[45] Gioia G, Isquith P, Guy S, Kenworthy L (2017). Behavior Rating Inventory of Executive Function 2 | BRIEF2. Hogrefe.

[46] Bruni O, Ottaviano S, Guidetti V, Romoli M, Innocenzi M, Cortesi F, Giannotti F (1996). The Sleep Disturbance Scale for Children (SDSC). Construction and validation of an instrument to evaluate sleep disturbances in childhood and adolescence. J Sleep Res.

[47] Kessels RPC, van Zandvoort MJE, Postma A, Kappelle LJ, de Haan EHF (2000). The Corsi Block-Tapping task: standardization and normative data. Appl Neuropsychol.

[48] Reynolds (2019). CTMT2 Comprehensive Trail Making Test, 2nd Edition.

[49] Lingjaerde O, Ahlfors UG, Bech P, Dencker S, Elgen K (1987). The UKU side effect rating scale. A new comprehensive rating scale for psychotropic drugs and a cross-sectional study of side effects in neuroleptic-treated patients. Acta Psychiatr Scand Suppl.

[50] Lemmens JS, Valkenburg PM, Peter J (2009). Development and validation of a game addiction scale for adolescents. Media Psychol.

[51] Lloret Irles D, Morell Gomis R, Marzo Campos JC, Tirado González S (2018). Spanish validation of Game Addiction Scale for Adolescents (GASA). Aten Primaria.

[52] Fenollar-Cortés J, Calvo-Fernández A, García-Sevilla J, Cantó-Díez TJ (2016). La escala Strength and Difficulties Questionnaire (SDQ) como predictora del TDAH: comportamiento de los índices SDQ respecto a las dimensiones “hiperactividad/Impulsividad” e “inatención” en una muestra clínica. An Psicol.

[53] Menéndez-García A, Jiménez-Arroyo A, Rodrigo-Yanguas M, Marin-Vila M, Sánchez-Sánchez F, Roman-Riechmann E, Blasco-Fontecilla H (2022). Internet, video game and mobile phone addiction in children and adolescents diagnosed with ADHD: A case-control study. Adicciones.

[54] Castellanos FX, Sonuga-Barke EJ, Milham MP, Tannock R (2006). Characterizing cognition in ADHD: beyond executive dysfunction. Trends Cogn Sci.

[55] Gross JJ (2002). Emotion regulation: affective, cognitive, and social consequences. Psychophysiology.

[56] Gross JJ (2015). Emotion regulation: current status and future prospects. Psychol Inq.

[57] Hoza B, Pelham WE, Milich R, Pillow D, McBride K (1993). The self-perceptions and attributions of attention deficit hyperactivity disordered and nonreferred boys. J Abnorm Child Psychol.

[58] Brilliant TD, Nouchi R, Kawashima R (2019). Does video gaming have impacts on the brain: evidence from a systematic review. Brain Sci.

